# A cost-effective smart metering approach towards affordable deployment strategy

**DOI:** 10.1038/s41598-023-44149-9

**Published:** 2023-11-09

**Authors:** P. Ezhilarasi, L. Ramesh, P. Sanjeevikumar, Baseem Khan

**Affiliations:** 1 Electronics & Communication Engineering, Dr. M.G.R. Educational, and Research Institute, Chennai, 95 India; 2 Electrical and Electronics Engineering, Dr. M.G.R. Educational and Research Institute, Chennai, 95 India; 3 University of South-Eastern Norway, Notodden, Norway; 4 Department of Electrical and Computer Engineering, Hawassa University, 05 Hawassa, Ethiopia

**Keywords:** Electrical and electronic engineering, Energy infrastructure

## Abstract

Revamping the power grid into a smart grid and modernizing it with advanced metering infrastructure are essential steps in addressing ongoing energy challenges. Smart meters play a pivotal role in power grid modernization by providing real-time energy-related data which fuels the control activities of modern grid. While the advantages of smart meters are evident, their deployment necessitates a comprehensive redesign of the grid architecture, involving smart end devices for monitoring and communication networks for efficient data exchange. Yet, achieving cost-effective and widespread adoption of these technologies poses a challenge, particularly in developing and underdeveloped nations due to high capital costs, technological constraints and uneconomical deployment strategies. Moreover, the prevailing research often advocates a complete transition to new smart meters to achieve 'smartness,' neglecting the potential of existing metering infrastructure upgrades. To address these concerns, this study proposes and simulates the design of a low-cost Smart Network Meter. Notably, this meter upgrades the existing meter infrastructure while validating a cost-effective deployment strategy. Furthermore, a consumer opinion survey was also conducted to compelling evidence supporting the adoption of the proposed low-cost smart metering solution.

## Introduction

The ongoing digitization and integration of Information Technology (IT) are driving transformative changes across various sectors. In the power sector, these technologies are being employed to effectively manage and regulate the power grid in real-time^1,2^. Consequently, the concept of the smart grid has emerged to monitor and administer the in near real time. This involves modernizing the power infrastructure, incorporating intelligent components and communication networks to mitigate the limitations of traditional power grids. Central to the smart grid framework is the Advanced Metering Infrastructure (AMI), a crucial component that enables remote metering and real-time monitoring through bidirectional communication^3^. The fuel of the AMI ecosystem is real-time data, which is actively collected by the smart meter integrated at the consumer's end. The smart meter core component of AMI gathers real-time data and collaborates with the AMI to transmit this information to utility providers. This dynamic linkage significantly enhances the management of the grid.

The smart meter's capability drives the shift from conventional to smart metering processes, providing numerous benefits to consumers, utility providers, and societies, both directly and indirectly. This transition occurs through various AMI deployment projects and schemes on a global scale. However, despite their rapid implementation, smart meters encounter challenges^4^ such as lack of standardization, varying design specifications, interoperability issues with AMI ecosystem components, extended deployment periods, security and privacy concerns. Notably, the cost associated with modifying existing infrastructure significantly influences the acceptance and penetration rate of smart meters among consumers^5^. Interestingly, the majority of global smart meter research has predominantly centered around data analytics, communication infrastructure, and data management algorithms, with less emphasis on the cost-effectiveness of utilizing existing metering infrastructure.

**Figure 1:**
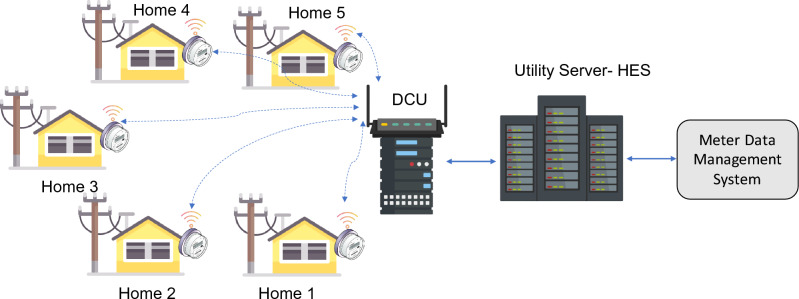
Common smart metering architecture.

The transition from a conventional power grid to a smart grid mandates a thorough overhaul of both infrastructure and architectural design. It's imperative to ensure the sustainability of this transformation, especially for implementation in economies with lower resources^6^. Within this context, achieving an affordable smart metering system demands meticulous attention to the design of smart meters and the formulation of deployment strategies. In the existing smart metering framework, residential consumption data is transmitted to utility providers through diverse devices, contingent upon geographical conditions and energy policies of various countries^7^. Foremost, consumption data from residential users is initially channelled to nearby Data Concentrator Units (DCUs). Subsequently, this data is aggregated within the Head End System (HES) of the utility provider, then forwarded to the Meter Data Management System (MDMS). The commonly used existing smart metering architecture is illustrated in Fig. 1.

As a consequence, this paper introduces the concept of a Smart Network Meter (SNM) to mitigate the substantial costs associated with smart meter design and deployment strategies. The SNM presents a pragmatic solution for clusters of houses by enabling smart metering without necessitating the replacement of existing digital meters at the consumer end. The transformation of a digital meter into a smart one is achieved by connecting an add-on device, which extracts consumption details and communicates them wirelessly to the central SNM. Furthermore, the data processing algorithm for smart metering in SNM follows a decentralized framework that does not rely on a central utility server for all smart metering tasks.

The proposed SNM is meticulously simulated in Proteus to validate its viability and assess its efficacy in smart meter functionalities. The details are reported in Section “Smart network meter”. The simulation results clearly indicate that the functionality of the proposed SNM can effectively address the challenge of deployment costs associated with smart meters, particularly in developing and underdeveloped countries.Additionally, a survey of consumer opinions was conducted, and the results provide valuable insights into the acceptance of the SNM among residential consumers. These findings are detailed in Section “Justification of the SNM through a consumer opinion survey” of this paper, complementing the argument for the adoption of SNM.Furthermore, to justify the cost-effectiveness of the SNM, a comparative analysis is conducted between the existing and proposed systems. The results of this analysis are discussed in Section “Comparative cost analysis”.

## Literature review

The requirement for electricity has consistently risen since its discovery. As a result, the importance of using electricity efficiently has become clearer, leading to the invention of devices to measure energy usage. Across time, various forms of energy measuring tools have been developed. Among these, the analog meter endured as a prominent design for a substantial duration and is still in use in certain nations for measuring purposes. With the emergence of the digital age, analog meters were progressively substituted by their digital counterparts. Pushing this transformation ahead, digital meters are now being superseded by smart meters, driven by technological progress.

Implementing smart meters encounters a range of hurdles in each nation, influenced by technological and societal elements. One of the most prevalent issues is the cost tied to smart meter deployment, as well as establishing communication infrastructure and managing upkeep^8^. Another notable difficulty pertains to consumer approval, as the potential merits of smart meters remain unfamiliar to consumers. While smart meters cut operating cost for utility companies in contrast to traditional meters, they impose a financial load on utility providers directly^9^ and consumers indirectly^10^. When considering the replacement of their old meters with smart meters, consumer’s willingness becomes uncertain due to the substantial cost involved and the less apparent benefits they perceive^9^. In light of these aforementioned challenges, creating an economically viable smart meter that offers all the functions of smart metering is crucial. Furthermore, devising a deployment strategy that doesn't mandate overhauling the existing digital metering system is essential.

The smart metering system has undergone extensive research and significant enhancements in the last decade. Within this domain, research articles on smart meters have produced a wide array of findings, addressing diverse concerns. For the purpose of streamlining this literature review, emphasis has been placed on articles that explore the costs linked to the design of smart meters. To achieve this objective, a meticulous approach was employed to gather prior research articles pertaining to smart meters from sources like Web of Science and Scopus. Advanced search queries were constructed, utilizing criteria such as (('Document Title & Find article with these keyword': ("affordable" OR "cost-effective" OR "low-cost") NOT ("energy cost" OR "power cost" OR "water")) AND ('Document Title': smart meter -data)). Furthermore, through a rigorous filtration process, research articles that do not fit within the defined scope are systematically excluded. Subsequently, design-focused papers are chosen from the gathered research articles, and their design architectures are thoroughly examined.

Abate et al.^11^ proposed a cost-effective smart meter for IoT applications, featuring two metering algorithms. The authors used an STM32F2 microcontroller, a wM-Bus radio module operating at 169 MHz (to connect smart meter to IoT ecosystem), and a CAN bus interface to develop a prototype of the smart power meter. Authors concluded that the developed prototype is cost effective by using low-cost design components such the shunt resistor (2€), the ADE7913 (around 10 €), current and voltage transducer (almost one 100 €). And also, author justified low cost by sharing the wM-Bus infrastructure of smart water and gas meter. Similarly, Labib et al.^12^ developed a low-cost smart meter with demand-side load management by incorporating affordable components into its design.Moreover, the authors compared the total cost of implementing the proposed smart meter ($40 USD), which is significantly lower compared to smart meters available in the market. Ewerton de Sousa et al.^13^ developed a smart meter with a complete set of low-cost components to measure and monitor electrical parameters, with an approximate total cost of $82.77.

**Figure 2:**
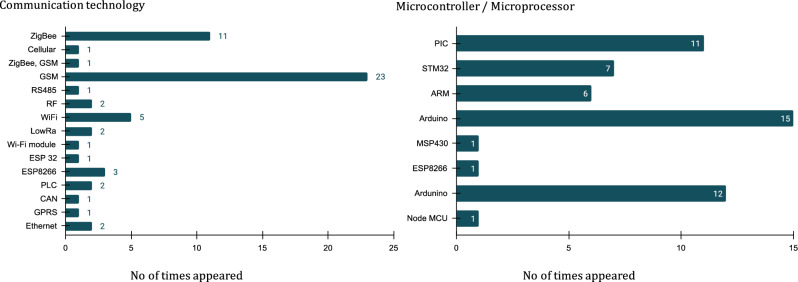
Analysis of smart meter design-based research articles.

In another study^14^, Edgar Saavedra et al. developed a low-cost, self-powered smart meter for challenging environments, which cost around 80 € initially, with the potential for the device cost to be lowered to 20–30 € through chain production. Furthermore, another low-cost smart meter, named OpenZmeter, was developed by Eduardo Viciana et al.^15^, with an assembly cost of less than 50 USD. From the aforementioned literature, it is evident that achieving a low cost for smart meters is primarily accomplished through the selection of affordable components. Furthermore, all of these works necessitate the replacement of existing metering infrastructure with new smart meters. Further the collected research paper was analyzed for the components selection to develop smart meter design. The results are visualized in Fig. 2 based on the frequency of their appearance in the research articles. According to the analytical study, Arduino controllers and GSM communication stand out as the most frequently mentioned topics in the research articles. Furthermore, the collected research papers were analyzed to determine the components selected for developing smart meter designs. The results are presented in Fig. 2, illustrating the frequency of appearance of controllers and communication modules in the collected research articles. According to the analytical study, Arduino controllers and GSM communication emerged as the most frequently mentioned topics in the research articles. Following these, ZigBee occupied the next position in the context of low-power and short-range communication for smart metering.

One primary issue with existing smart metering technology is the costly installation of new smart meters in every household. A significant portion of smart meter research underscores the implementation of new smart meters rather than leveraging the preexisting metering infrastructure already present in households. Notably, there exists limited previous evidence regarding the use of existing metering systems in smart metering endeavors to attain a more economical deployment. Conversely, retrofitting is viewed as a cost-efficient strategy, particularly for developing and underdeveloped nations. This becomes especially pertinent when considering factors like deployment duration, investment costs, and the rate of smart meter adoption. Tewolde et al.^16^ developed a laboratory-based low-cost retrofit module for gas meters. This module consists of a low-cost, high-resolution mechanical encoder that can be retrofitted onto an existing gas meter. Similarly, Nelson Pimenta et al.^17^ developed a smart water meter by retrofitting mechanical water meters to enable automatic meter readings. These retrofitting methods avoid the need for potential investments in new hardware architecture for the intended meter development. In alignment with the concept of retrofitting for smart energy meters, Ezhilarasi et al.^18^ proposed a Smart Network Meter (SMN) model for low-cost design without the need to replace existing metering infrastructure. This prior work by the authors of this paper is supported by a detailed literature review for justification.

The Sustainable Development Goals (SDGs) lay out a vision for a more sustainable and improved future for everyone. Notably, SDG-7.b places emphasis on enhancing infrastructure and modernizing technologies to offer clean energy services that are both sustainable and affordable. In alignment with this overarching vision, the focus of this paper revolves around simulating the SNM system and validate the viability for proposed design in real time implementation. To accomplish this, the authors utilized the Proteus software to execute simulations based on a previously established model of SNM^18^, ensuring its practicality. The outcomes of these simulations are detailed in Section “Smart network meter”. Furthermore, to gain insight a consumer opinion surveys were conducted to assess consumer’s willingness to adopt SNM, and the results are discussed in the following section. To further substantiate the cost-effective smart meter deployment strategy, this paper analyzes three distinct case studies and presents their results in Section “Comparative cost analysis”.

## Justification of the SNM through a consumer opinion survey

While smart meters offer numerous benefits to consumers and utility providers, their acceptance remains uncertain in developing and underdeveloped countries^5,19^. Consequently, a residential consumer opinion survey was conducted among to validate the low-cost deployment of proposed SNM. The survey questions are designed with simplicity and clarity, considering that the targeted respondents are general residential consumers. A preliminary survey involving ten respondents was initially conducted to refine the questionnaires for both ease of completion and accuracy. Subsequently, modifications were made to the questionnaires based on the feedback received.

The final survey was conducted online and gathered responses from 2111 participants across India. To achieve a 95% confidence level and a margin of error of ± 2.9%, a sample size of 1614 was determined to be necessary. Collecting 2111 responses, which is more than the required sample size, affirms the accuracy of the survey's results. The objective of this survey is to elucidate the concepts of smart meters and SNM to consumers and to gain insights into their preferences.

Key survey questions and their corresponding results are detailed in the following section. Regarding the question about interest in real-time energy monitoring, it was observed that 1937 respondents (91.8%) expressed interest in learning about real-time energy consumption details. While explaining the operation of the smart meter, 980 respondents (46.4%) indicated their comprehension of its purpose and functioning. Impressively, smart meters garnered positive reception from consumers, with 70.3% (1485 respondents) expressing their willingness to embrace them, in contrast to 29.7% (626 respondents) who expressed reluctance. This statistical data underscores the respondent’s keen interest in availing the benefits associated with smart meters.

**Figure 3:**
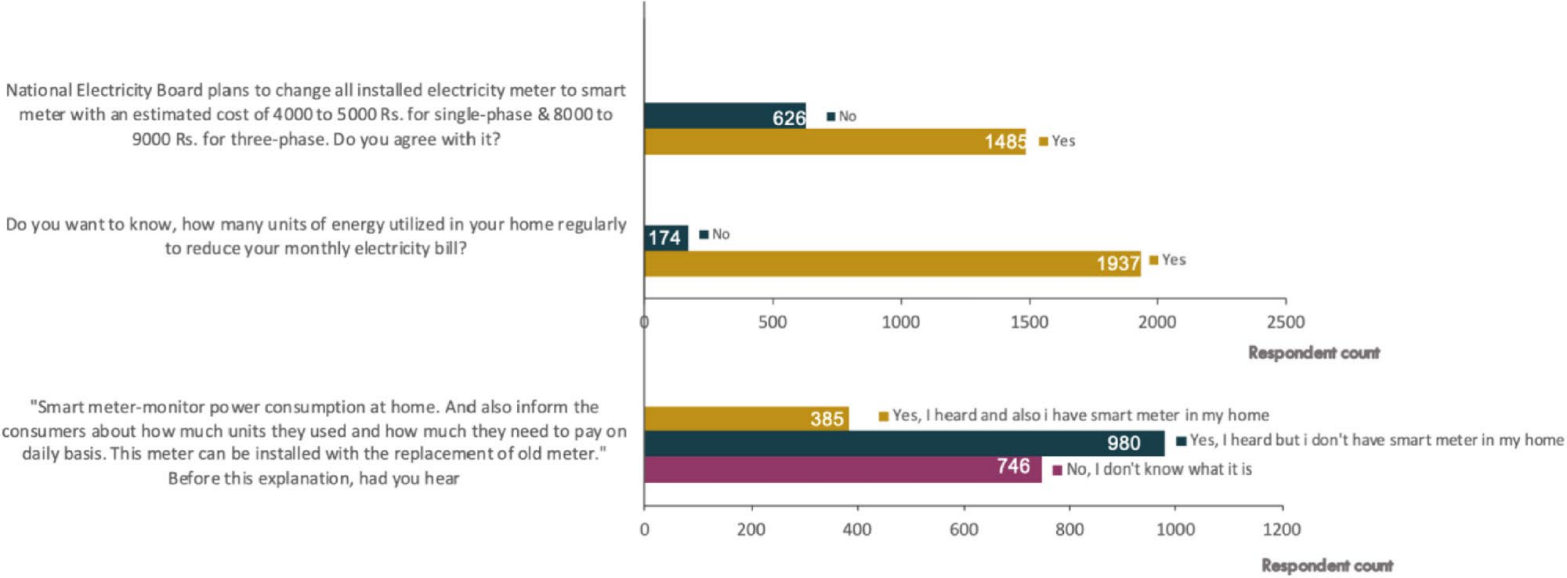
Survey responses—1.

Participants were provided with an explanation of smart meters and then asked, 'Had you heard of smart meters before this explanation?' This question aimed to assess the participants' familiarity with and prior understanding of smart meters. Among the respondents, 46.42% (980) reported that they know about smart meters but do not have one in their household, while 35.33% (746) stated that they do not know what a smart meter is. In contrast, 385 respondents (18.25%) indicated that they have a smart meter installed in their homes. The aforementioned responses are illustrated in the Fig. 3.

**Figure 4:**
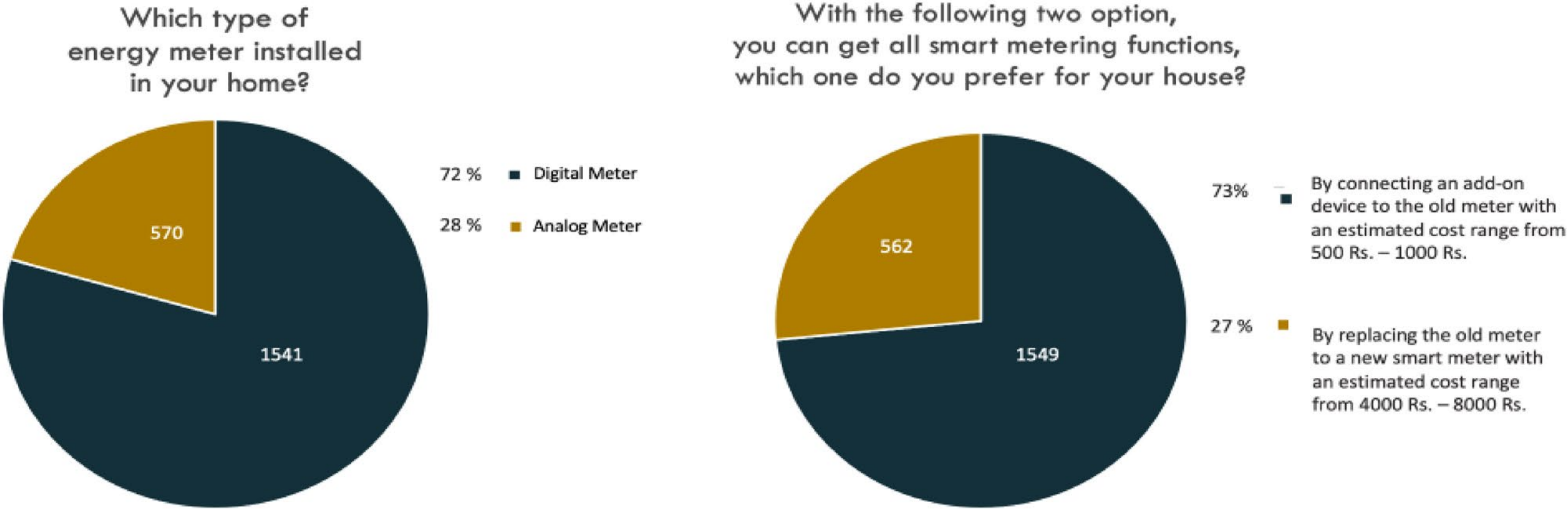
Survey responses—2.

Furthermore, respondents were presented with a question about the type of energy meter installed in their households, and the results are depicted in Fig. 4. From the received responses, it is observed that 1541 respondents (72% of households) possess digital meters for reading purposes, rather than analog meters. This level of digital meter penetration suggests that SNM offers a feasible approach to smart meter implementation by integrating an add-on device with the existing digital meters.

When respondents were presented with cost details of both the existing and proposed SNM, a notable trend emerged. Out of the 2111 respondents, 1549 individuals expressed a preference for upgrading their digital meters using the add-on device offered by SNM (costing between 1000 to 1500 INR) rather than acquiring an entirely new smart meter (costing 3000 to 4000 INR). The trend in respondent preferences illustrates a strong inclination towards adopting the add-on device as opposed to obtaining an entirely new smart meter, primarily driven by cost constraints. Notably, 73% of the surveyed respondents expressed their willingness to incorporate the add-on device for upgrading their existing digital meters to smart meters, as facilitated by the proposed SNM and results are presented in the Fig. 4.

## Smart network meter

### Architectural comparison of existing smart metering technology and proposed SNM technology

The evolution of metering methodologies has evolved alongside technological advancements since the inception of electricity meters. Concurrently, the design of metering devices has adjusted to scientific progress, transitioning from conventional to digital meters to accommodate changing needs. In the past, energy consumption readings relied on human intervention, requiring manual recording by a workforce. However, the advent of smart metering technology has fundamentally transformed this process, eliminating human involvement and streamlining the time needed for bill preparation. The implementation of smart meters at consumers' premises has enabled real-time energy consumption monitoring and metering.

In recent times, the widespread adoption of smart metering typically involves the installation of new smart meters in households. These smart meters collect consumer data, which is subsequently aggregated and stored within nearby Data Concentrator Units (DCUs). Data transmission occurs through Local Area Networks (LANs), utilizing diverse communication technologies such as ZigBee, Wi-Fi, Radio Frequency (RF), and Power Line Communication (PLC). Subsequently, the DCU communicates with utility servers via Wide Area Network (WAN) technologies like Global System for Mobile Communications (GSM) and General Packet Radio Service (GPRS) for further data processing.

**Figure 5:**
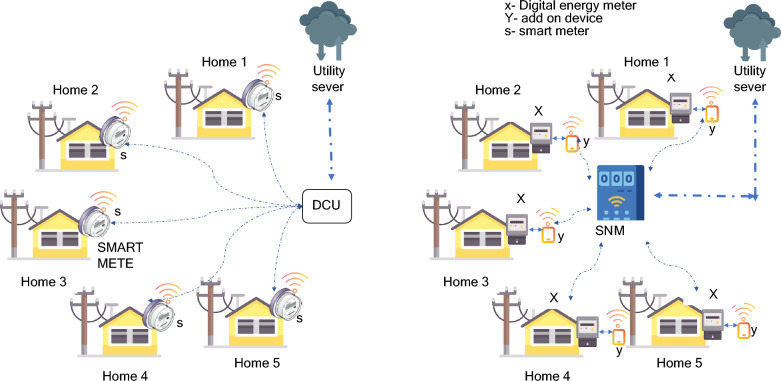
Architectural comparison of existing and proposed smart metering technology.

On the contrary, the Smart Network Meter (SNM) architecture presents an innovative approach. By introducing a single add-on device at the consumer's end, the existing digital energy meter can undergo an upgrade to a smart meter without requiring complete replacement. SNM possesses the ability to monitor a cluster of houses and its capacity determined by the microcontroller's capabilities. During the SNM design phase, energy consumption data is extracted from digital meters through the serial communication port (RS485) and conveyed to the central SNM for subsequent processing using a Zigbee transceiver. This transceiver at SNM receives the transmitted data from the add-on device, enabling it to gather data from all houses within the designated cluster. Afterward, SNM processes the collected data, offers real-time responses, and forwards the data to the server upon receiving a request. A visual depiction of the comparative architectural distinctions between the existing and proposed SNM smart metering systems is depicted in Fig. 5 and elaborated on extensively in Table 1.

**Table 1:** Comparison of existing smart meter and SNM.

Existing smart meter	SNM
Need to replace the old digital meter with the new smart meter for smart metering	Upgrade the existing digital meter by adding an add-on device for smart metering
Deployment cost is high since all houses fitted with new smart meters	Deployment cost is less since the cluster of houses is connected to one common SNM
Maintenance and firmware updates are carried out separately	Maintenance and firmware updates are carried out only at SNM

### SNM design and simulation

**Figure 6:**
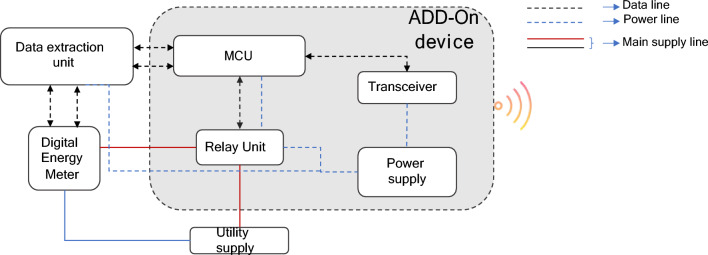
Add-on device block diagram.

The add-on device stands as an essential element of the proposed SNM system, encompassing five key functional blocks: data extraction, MCU, relay, communication, and power supply. Efficient extraction of data from digital meters is achieved through data extraction units, accessing specific registers as indicated in the manufacturer's data sheet. The MCU functions as the central unit responsible for data processing and communication with the SNM. The transceiver facilitates bidirectional communication, enabling data exchange, and is configured in a star topology, with the SNM serving as the master and the add-on devices functioning as slaves. The inclusion of relays enables remote supply control, responding to commands from the SNM. A dedicated power supply ensures uninterrupted device operation. This comprehensive add-on device design simplifies the process of transitioning to smart meters through the utilization of the SNM. The add-on device design's architecture is depicted in Fig. 6.

**Figure 7:**
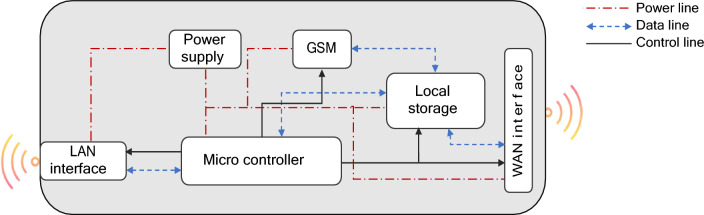
SNM design block.

The SNM encompasses components such as the Central MCU, LAN/WAN interfaces, local storage, GSM module, and power supply. Data from add-on devices is gathered via LAN using master–slave programming at the SNM. Following this, the acquired data is stored within a local storage unit. The MCU processes this data to execute smart metering functions, including remote metering, remote monitoring, remote supply connect/disconnect, and consumer notifications. Ultimately, the processed data is conveyed to the utility provider's server through GSM technology. The design structure of the SNM is depicted in Fig. 7.

The choice of communication is pivotal in determining the cost-effectiveness of smart deployment, as the cost associated with communication infrastructure directly impact the overall smart meter deployment costs. The suggested model entails three distinct communication networks: digital meter ↔ add-on device, add-on device ↔ SNM, and SNM ↔ Utility server. The digital meter employs a serial communication mode to interface with the add-on device, extracting energy consumption data from the digital meter's holding register.

**Figure 8:**
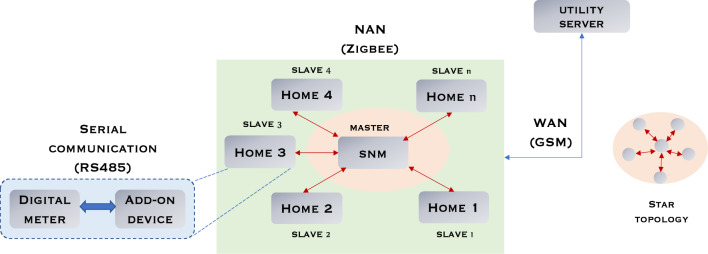
Proposed communication networks and topologies.

The add-on device is subsequently linked to the central SNM through a Near Area Network (NAN), enabling data and control signals to be exchanged between them. Following this, the processed data is transmitted to the utility server via a Wide Area Network (WAN) due to the need for data to traverse longer distances to reach the server. Within the proposed model, serial communication (typically RS485) is selected for data extraction due to its heightened security when compared to wireless protocols. For the NAN, the ZigBee protocol is adopted in a star topology, primarily for its cost-effectiveness and low power consumption. This protocol is also widely recognized in literature as a preferred choice for NAN communication in smart meter designs. The star topology allows all add-on devices (slaves) to establish direct connections with the SNM (master) through a master–slave handshaking mechanism. Ultimately, GSM (cellular communication) is opted for WAN due to its prevalence as a smart meter communication protocol for long-range communication. This choice leverages existing cellular communication infrastructure, significantly reducing associated communication infrastructure costs. The intricate communication network of the proposed metering system is depicted in Fig. 8.

### SNM simulations and results

This study utilized Proteus EDA for simulating the SNM, creating a practical testing environment that incorporates integrated microcontroller support. The simulation process involved designing and integrating sub-circuits for the proposed metering system, leading to the successful validation of most functionalities. However, Proteus's limitation in supporting wireless communication simulation necessitated the connection of COM ports on the computer to the TX and RX of the intended model. To realize the wireless communication between the Zigbee transmitter and receiver, the Virtual Serial Port Emulator was employed to establish a connection between the computer's COM ports. The SNM smart metering system is categorized into two operational models: Home model and SNM model.

The home simulation model consists of interconnected components: a load model, a separate digital meter model, and an add-on device model. Given the unavailability of dedicated load and digital meter models in the Proteus library, we developed these two models independently and later integrated them with the add-on device model. To establish the load model, we devised two configurations: one for a single-bedroom (1 BHK) and another for a double-bedroom (2 BHK) load model. This was achieved by selecting a combination of inductive and resistive loads from the Proteus library. This strategic selection enabled us to replicate a diverse array of appliances commonly encountered in real-world scenarios, including fans, lights, air conditioners, and other devices. The digital meter model encompasses sensors, a real-time clock module, a relay, and an Arduino Uno microcontroller. The Arduino controller employs a current sensor to measure current consumption, while a standard voltage value of 230 V is employed. By utilizing readings from the current sensor in conjunction with the voltage value and time data, real-time energy consumption details are calculated. The add-on device model incorporates an Arduino Uno, a relay, and a Zigbee module to facilitate its designated operation.

The SNM is powered by an Arduino Mega, functioning as its controller, alongside components including Zigbee (in the role of a coordinator), an SD card (for local storage), and GSM modules. Zigbee modules configured as end devices within household add-on devices, while the SNM's Zigbee module is configured as the coordinator. The Arduino Mega processes incoming data from add-on devices, subsequently storing it on the SD card for future purpose. The SNM incorporates a decentralized data processing framework to execute smart metering for clusters of houses. Within this framework, all smart metering functionalities pertaining to consumers are managed by the SNM itself, eliminating the dependency on the utility server. Only the data essential for grid control and operation is transmitted to the utility provider's server (in simulation virtual serial COM port) using the GSM module. The programming within the SNM manages tasks such as remote monitoring, control operations, and consumer notifications. In order to assess the performance and viability of the proposed architecture, five distinct home models were created, each featuring varying load conditions. Rigorous simulation tests were undertaken to validate the functionalities of remote metering, remote control, and consumer notifications.

Once the connection between the SNM and add-on devices was established in Proteus, the operational hex code files containing algorithms for both the SNM and add-on devices were uploaded into the Proteus sketch. The programming codes were initially formulated and tested using the Arduino Integrated Development Environment (IDE), an open-source software designed for programming Arduino boards. When the Arduino IDE program is compiled, it generates a hex file that can be utilized to simulate interconnected devices within the Proteus platform. The algorithm necessary to encompass the functionalities mentioned is outlined as follows:

Stage 1: Data read by the digital meter is extracted and sent to the add-on device controller unit.
Stage 2: The received data is communicated to the SNM through the Zigbee module.
Stage 3: Decentralized data processing is done on received data to carry out the smart metering.
Stage 4: From SNM, the processed data is then sent to the utility server via WAN communication technologies.

**Figure 9:**
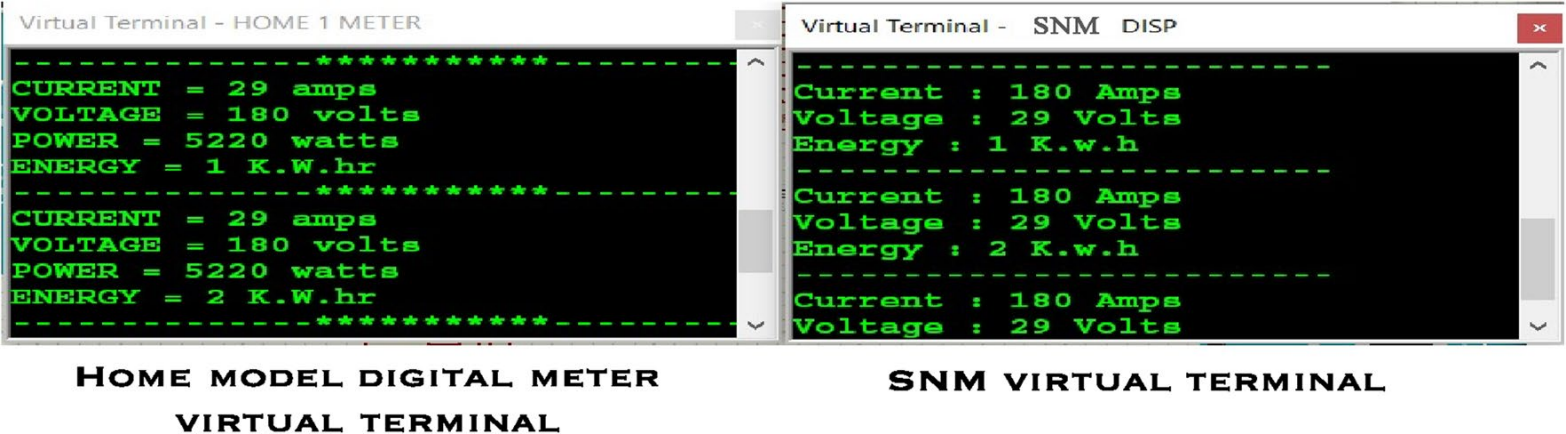
Remote metering result.

Following the establishment of a successful simulation environment for SNM development, comprehensive tests were undertaken to validate the functionalities of remote metering, remote monitoring, remote supply connect/disconnect, and consumer alerts. To verify remote metering, the virtual terminal value of the digital meter within the home model is compared with the virtual terminal value of the SNM. This comparison determines whether the readings from both sources match or not. The obtained results illustrated in Fig. 9 shows the SNM’s efficiency in remote metering.

The functionality of remote supply connect/disconnect was executed, and the outcomes were documented in a Table 2. This feature was implemented to respond to the consumer's payment status, generating a corresponding control signal from the SNM. This signal is then transmitted to the add-on device, instructing it to either connect or disconnect the relay unit accordingly.

**Table 2:** Remote supply connect/disconnect.

Command initiated from the SNM	Consumer ID	Supply status	Billing status	Signal to relay	Supply status after decision
YES	Home 1	Connected	not paid	Low	Disconnected
NO	Home 2	Connected	Paid	No action	Connected
YES	Home 3	Disconnected	Paid	High	Connected
YES	Home 4	Connected	Paid	No action	Connected
YES	Home 5	Connected	Not paid	Low	Disconnected

**Figure 10:**
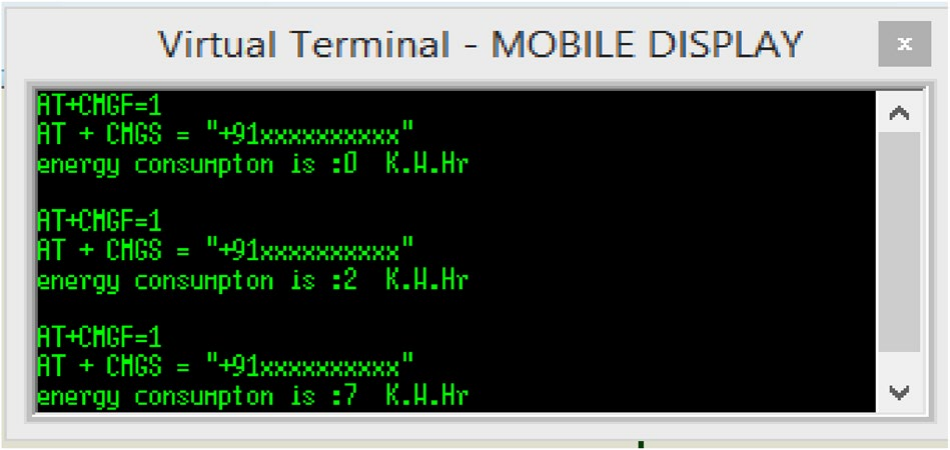
Consumer alert notification.

Regarding the functionality of consumer alert notifications, the GSM module is responsible for delivering notifications. Whenever the consumer's mobile device (in simulation—virtual serial port) sends a request to trigger, the SNM responds by updating the consumer's mobile device with energy consumption details. This outcome is depicted in Fig. 10.

**Figure 11:**
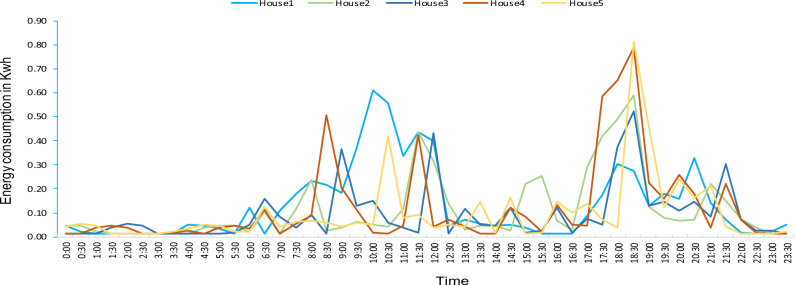
Five-house energy consumption for 24 h.

After completing the initial round of testing, an additional test was carried out to monitor 24-h energy consumption in 30-min intervals for five houses. In this test, the digital meter of each house was programmed with preloaded values, encompassing both energy consumption and time, to accurately simulate real-world conditions. The primary objective of this test was to assess the efficiency of the SNM under dynamic load conditions. The outcomes of this test have been visually represented in Fig. 11.

## Comparative cost analysis

Cost is a pivotal factor in all forms of research and innovation, as it determines the feasibility of real-time processing at a larger scale. This aspect significantly contributes to the efficiency of the planning process and production. The modernization of existing grids necessitates significant infrastructural alterations and substantial investments, along with maintenance cost. The cost associated with advanced metering infrastructure includes the cost of smart devices (such as smart meters, sensors, Remote Terminal Units, etc.), communication infrastructure, software for data processing, and server upkeep. Indirectly, consumers bear the costs of smart meters, a factor contributing to the lower adoption rates in economies with limited financial resources. Additionally, the costs related to the widespread deployment of smart meters can surge during large-scale implementation. To assess the cost-effectiveness of the SNM, a comparative analysis is conducted across three case studies, each evaluated under two distinct scenarios: Scenario 1 (SNM system) and Scenario 2 (existing smart metering system). The case studies includes the following scenarios: A. Houses in a Flat, B. Houses in Streets, and C. Houses in a Village. These case studies are analysed in two scenarios: with the existing smart meter infrastructure and with the SNM. The specifics of this analysis are discussed in the subsequent section.

To facilitate a comprehensive cost comparison analysis, the cost of an existing smart meter, including its installation, is sourced from the study titled "AMI rollout strategy and cost–benefit analysis for India"^3^. Conversely, the cost of SNM board assembly and add-on devices is derived from actual market prices. The communication range of the Zigbee transceiver spans from 10 to 100 m within the line of sight. Beyond this range, Zigbee repeaters are employed to ensure effective communication with the SNM. Furthermore, a single repeater can accommodate communication from up to 32 devices. The number of consumers connected to the SNM is calculated based on the board assembly capacity of the Arduino controller. For the Arduino Mega and Arduino Uno, 10 and 5 consumers are respectively allocated, ensuring interference-free communication with each consumer through the employment of both hardware and software serial communication ports.

The overall cost, denoted as ${C}_{T}$, for the deployment of smart meters is characterized by the amalgamation of several components. These include the unit cost of smart meters ${C}_{M}$, their installation cost ${C}_{I}$, the cost of repeaters ${C}_{R}$, and the cost of the add-on device ${C}_{A}$. In order to perform costanalysis, parameters and elements linked to both the existing smart metering technology and the SNM technology are adjusted to conform to specific constraints. These adaptations are made to accurately determine the total cost of deployment.In the context of SNM metering system, the presumed constraint guides the division of the total number of houses into clusters, determined by the capacity of the SNM board assembly. Subsequently, each cluster is chosen, and the coverage area of the ZigBee transceiver is evaluated. If the coverage area extends beyond 100 m, the metering architecture incorporates repeaters at intervals of 100 m to ensure seamless communication with the SNM.

The mathematical representation of the total cost within the SNM metering scenario (scenario 1) is formulated as indicated in Eq. (1).

1 $${C}_{Ts1}=(\left(m*{C}_{R}\right)+\left(\sum_{i=1}^{q}\left({C}_{SNMm}+{C}_{I}\right)+\sum_{j=1}^{r}\left({C}_{SNMu}+{C}_{I}\right)\right)+({C}_{A}*n))$$

where, q and s derived by dividing n by 10, m = number of repeaters, n = total number of consumers and $n\ge 10$; q = quotient of $\frac{n}{10}$ and $q>0$ = number of Arduino Mega-based SNM, d = remainder of $\frac{n}{10}$, r = quotient of ${\frac{d}{5}|}_{5\le d<10} and r>0$ = number of Arduino uno-based SNM, ${C}_{SNMm}$= cost of Arduino mega-based SNM, ${C}_{SNMu}$= cost of Arduino uno-based SNM, ${C}_{Ts1}$= total cost for scenario-1, ${C}_{Ts2}$= total cost for scenario 2, Coverage area = $\pi *{radius}^{2}\approx 314 {meter}^{2}$, Radius = 100 m since the coverage area for the ZigBee communication is 100 m with a line of site. From the market price, the add-on device cost ${C}_{A}$ = 500, ${C}_{SNMm}$ = 10,000 INR and ${C}_{SNMu}=\mathrm{3000}$ are estimated.

The mathematical equation total cost for exiting the metering scenario (scenario 2) is represented in Eq. (2).

2 $${C}_{Ts2}=\left(\sum_{i=1}^{n}\left({C}_{SM}+{C}_{I}\right)\right)$$

where, $${C}_{SM}={cost of exsisitng smart meter, C}_{A}=0 and {C}_{R}=0$$ for this scenario since smart meters are directly connected to the data concentrator.

### Algorithm to cost analysis mathematical model

I. Cluster the consumers based on the SNM communication coverage range.
II. Identify the number of consumers in each cluster n.
III. If consumers are more than 10, perform the division of n by 10 and store quotient as q and remainder as d.
IV. If $5<d<10$ → assign q = 1& r = 0; if $d\le 5$→ assign q = 0 & r = 1.
V. If consumers less than $5<n<10$→ assign q = 1& 1 = 0; if $n\le 5$ → assign q = 0, r = 1.
VI. Use the planned mathematical model to calcu1ate the total deployment cost with derived parameters in the previous steps.

**Figure 12:**
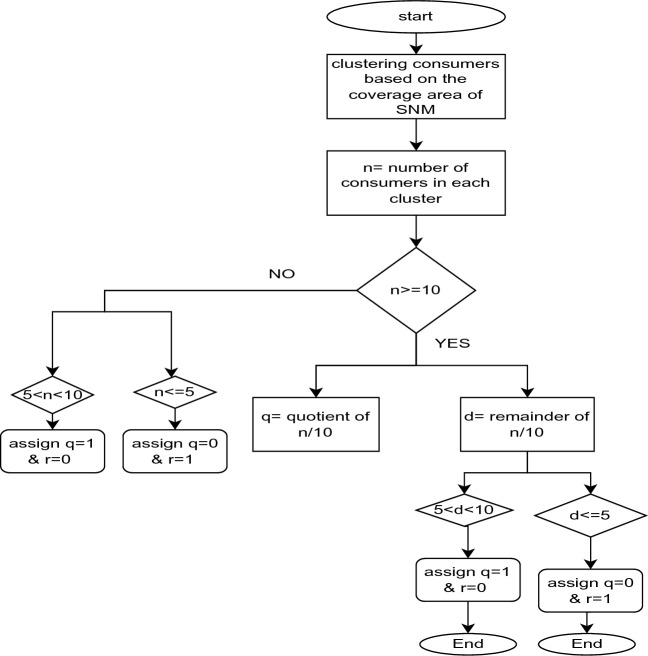
Mathematical modelling flow chart of cost analysis.

The process mentioned above is used to model the mathematical formula for comparative cost analysis and is illustrated in Fig. 12.

### Case A: houses in flat

In this case, a flat with 60 houses is identified near the Maduravoyal locality in Chennai, verifying that all houses are fitted with digital meters. For scenario 2, it is mandatory to change all digital meters to new smart meters with the existing meter technology. Therefore, 60 new smart meters are required to cover all 60 consumers from the flat for smart metering in the context of existing metering technology (scenario 2). Using ${C}_{SM}$ = 3500 INR and ${C}_{I}$ = 500 INR as considerations, and with Eq. (2), the cost for executing smart metering in the selected flat based on the existing smart meter technology is calculated as ${C}_{Ts2}$ = 2,40,000 INR.

**Figure 13:**
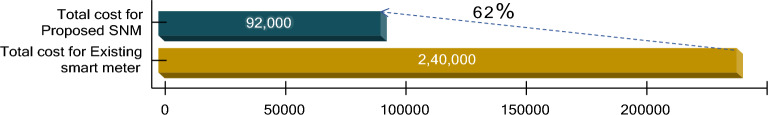
Case A cost analysis.

Regarding SNM, the coverage area should be considered before calculating the total cost and determining the requirement for ZigBee repeaters. Even though the flats are in high-rise buildings, in this case study, the distance from the ground determines the coverage of SNM rather than the area. The height of the flat is 600 feet, and the total number of consumers is 60 (n = 60); for every 100 feet, 10 consumers are equally distributed.From the equation, it's determined that 6 SNMs (q = 6), based on Arduino Mega, are required to cover 60 consumers without involving a repeater (m = 0) in the architecture. Due to the equal consumer density distribution, Arduino Uno-based SNM is not used in this case study (r = 0). The total cost for smart meter deployment is calculated using Eq. (1), considering the mentioned constraints.With the above constraints, the total cost ${C}_{Ts1}$=92,000 is calculated for scenario-1 using Eq. (2). The analysis reveals that the deployment cost for smart meters is reduced by 62% in SNM compared to existing meter technology, as depicted in Fig. 13.

### Case B: houses in street

This case covers residential consumers from a street with scattered locations selected rather than the closed location proximity scenario from case A, to validate the effectiveness of the proposed SNM. For this case, a street in the Mogappair East locality in Chennai is identified, and it's ensured that out of 40 consumers on that street, 35 consumers are equipped with digital meters.In scenario 2, 35 digital meters are changed to new smart meters using existing technology. Thus, 35 new smart meters are required to cover all 35 consumers (n = 35) on the street for smart metering in the context of existing metering technology (scenario 2). Using Eq. (2) and considering the constraints of this scenario, the total cost ${C}_{Ts2}$ = 1,40,000 is calculated to implement smart metering for this case study with existing metering technology.For the cost analysis in scenario 2, these 35 consumers are divided into clusters based on the coverage area of SNM. According to the geographical density of the consumers, three clusters are created, each containing 13, 7, and 15 consumers, respectively. The planned model evaluates each cluster using Eq. (1), and their results are aggregated to calculate the total cost output for this case study. The details of these clusters are reported in Table 3.

**Table 3:** Cost analysis for case B.

Cluster	n	q	r	m	${C}_{Ts1}$
1	13	1	1	0	20,500
2	7	1	0	0	14,000
3	15	1	1	0	21,500
	Total ${C}_{Ts1}$				56,000

This case covers residential consumers from a street with scattered locations selected rather than the closed location proximity scenario from case A—flats to validate the effectiveness of the proposed SNM. For this case, a street from the Mogappair east, Chennai locality is identified and ensured that out of 40 consumers from that street, 35 consumers are installed with a digital meter. For scenario 2, 35 digital meters are changed to new smart meters with existing technology. So, 35 new smart meters are required to cover all 35 consumers (n = 35) in the street for smart metering with existing metering technology scenario-2. From Eq. (2) and the constraints of this scenario, the total cost ${C}_{Ts2}=\mathrm{1,40,000}$ is calculated to implement smart metering for this case study with existing metering technology.

**Figure 14:**
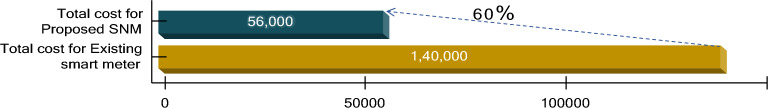
Case B cost analysis.

According to the cost model, 56,000 INR is required to cover 35 consumers from the selected street under smart metering with SNM, resulting in a 60% cost reduction compared to existing technology, as shown in Fig. 14.

### Case C: houses in village

Grid modernization becomes successful only when both urban and rural areas are under real-time monitoring. Consequently, cost analysis is also carried out in a village to validate the effectiveness of SNM. For this case, a village near Thiruverkadu, Chennai, is selected, comprising 346 houses. From these 346 houses, it is identified that 300 houses are equipped with digital energy meters. In scenario 2, 300 consumer meters were replaced with 300 new smart meters (n = 300) to implement smart metering with existing technology. The total deployment cost ${C}_{Ts2}$=12,00,000 for this case study is derived using Eq. (2). Unlike cases A and B in this study, houses are more scattered from each other in this scenario. According to the cost model, the 300 houses are clustered based on their coverage by the SNM. Due to geographical conditions, similar houses are grouped. There are 20 groups, each with 5 houses in a cluster; 4 groups, each with 6 houses; 6 groups, each with 10 houses; 6 groups, each with 8 houses; and 2 groups, each with 4 scattered houses. Using the planned model, each group is evaluated with Eq. (2), and the results are aggregated to determine the total cost output of this case study. Their details are reported in Table 4.

**Table 4:** Cost analysis for case C.

Cluster	n	q	r	m	${C}_{Ts1}$	Similarity count	Cumulative ${C}_{Ts1}$
1	5	0	1	0	6000	20	12,000
2	6	1	0	0	13,500	4	54,000
3	10	1	0	0	15,500	6	93,000
4	8	1	0	0	14,500	6	87,000
5	4	0	1	1	7500	2	15,000
						Total ${C}_{Ts1}$	2,61,000

**Figure 15:**
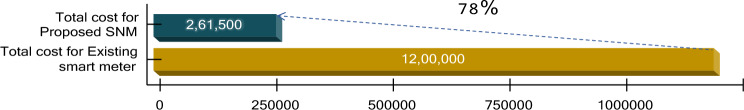
Case C cost analysis.

Since scattered houses are present in the last cluster, repeaters are used to complete the SNM communication. The total cost, C_Ts1 = 2,61,500 INR, is required for smart metering with SNM for all 300 houses in the village. Based on this analysis, a 78% reduction in deployment cost is achieved with the help ofSNM when compared to existing smart meter technology. These details are visualized in Fig. 15.

A detailed report on the cost comparative analysis for the three case studies is presented in Table 5. The report demonstrates that the proposed SNM deployment cost is reduced, ranging from 60 to 78% for the case studies. In case C, the percentage of cost reduction is higher when compared with the other two cases due to the utilization of more Arduino Uno-based SNMs. From the analysis, it's observed that the degree of cost reduction is mainly determined by the number of consumers within the coverage area of SNM and the type of SNM board assembly.

**Table 5:** Comparative cost analysis.

Case study	Total cost for proposed SNM technology (scenario-1)	Total cost for existing smart meter technology (scenario-2)	Percentage of cost reduction
A	92,000	2,40,000	62%
B	56,000	1,40,000	60%
C	2,61,500	12,00,000	78%

## Conclusion

This study presents the simulation of a cost-effective smart metering system employing the SNM approach within Proteus software. The scope of work includes the design and simulation of add-on devices for household and the SNM. The simulated SNM incorporated with decentralised data processing framework to perform smart metering without depending on utility server data. Notably, the development of SNM's functional codes is executed through the Arduino IDE. Moreover, to substantiate the viability of this approach, a consumer opinion survey was administered, affirming the efficacy of the proposed SNM in facilitating economical smart metering. To underpin the feasibility of this cost-effective deployment strategy, a comparative cost analysis was conducted with three distinct case studies, each representing different residential consumer types. The outcomes reveal a substantial cost reduction, ranging from 60 to 78%, across all three cases, validating the proposed SNM design as cost-effective and its deployment strategy as affordable when compared with existing system. Future investigations will focus on prototype development and real-time implementation of the proposed SNM in a real-world scenario to assess SNM’s cost-effectiveness in practical applications. Furthermore, the research direction will focus on reducing congestion in the communication network, drawing insights from this proposed SNM design.

## Experiments/surveys/questionaries

We authors confirm that all experiments were performed in accordance with relevant guidelines and regulations.

## Methods

We authors confirm that all methods were carried out in accordance with relevant guidelines and regulations.

We authors confirmed that the institutional committee approved the experiments and/or survey.

## Data Availability

The datasets used and/or analyzed during the current study available from the corresponding author on reasonable request.
